# Quality of life, Illness Perception, Self-perceived success, estimation of Depression/Anxiety symptoms and Disability Assessment, in adult with cerebral palsy

**DOI:** 10.1192/j.eurpsy.2023.1591

**Published:** 2023-07-19

**Authors:** E. N. Gruber, S. M. Biocina

**Affiliations:** 1 PC Sct Hans Roskilde, afdeling R, Roskilde, Denmark; 2 University Psychiatric Hospital Vrapce, Department of Social Psychiatry, Zagreb, Croatia

## Abstract

**Introduction:**

Recent studies is showed that adults with Cerebral Palsy (CP) have an elevated prevalence of mental health disorders, especially increased risk of depression or anxiety. Perceptions of the CP condition, and coping behaviors often affect the impact of the condition on the child with CP and his/her family.

Several studies have affirmed that some factors such as interpersonal relationships, sexuality, and physical conditions are also crucial to a higher QoL in the persons with CP.

A Danish study showed that 55% of Danish adults with CP (aged 29–35 years) were unemployed, did not cohabit with a partner and did not have children, compared with only 4% of the control population.

**Objectives:**

to show a case of a 50-year-old male person with cerebral palsy

**Methods:**

case study

The three functional classifications (GMFCS-E&R, CFCS and MACS) is used to provide functional description together with The Quality-of-Life Scale (QOLS), World Health Organization Disability Assessment Schedule 2.0 – (WHODAS-interview), Flourishing Scale Self-perceived success (FS), Depression Anxiety Stress Scales - 10 (DASS-10), the Brief Illness Perception Questionnaire (Brief IPQ)

**Results:**

male, 50 år

Quality of Life score: 90

Flourishing scale (FS): 47

Depression Anxiety Stress Scales: 9

the Brief Illness Perception Questionnaire (Brief IPQ):45

Communication issues: CFCS (Communication Function Classification System): Level I

Having a Partner: Domestic partner- reside together with partner, don’t have children. having af parents and brothers that are a great support

Type of Housing: Independent living (own housing, 1 hour of assistance per week)

Mobility issues: GMFCS (Gross Motor Function Classification System): Level II, MACS (Manual Ability Classification System): Level I

**Conclusions:**

Case is showing 50 years old male with cerebral palsy who has not an intellectual disability and who has a high life quality, high self-perceived success, moderate anxiety and high perception of illness. Social, family and romantic relationships together with leisure time and sustainable physical activity and exercise was emphasized.

**Disclosure of Interest:**

None Declared

